# Assessment of the Quality of COVID-19 Antigen Rapid Diagnostic Testing in the Testing Sites of Ekiti State, Nigeria: A Quality Improvement Cross-Sectional Study

**DOI:** 10.7759/cureus.24173

**Published:** 2022-04-16

**Authors:** Olufunmilola Kolude, Eyitayo E Emmanuel, Ayomide O Aibinuomo, Tope M Ipinnimo, Mary O Ilesanmi, John A Adu

**Affiliations:** 1 Emergency Preparedness and Response, World Health Organization (WHO) Field Office, Ado-Ekiti, NGA; 2 Community Medicine, Federal Teaching Hospital, Ido-Ekiti, NGA

**Keywords:** nigeria, testing sites, covid-19, antigen-rdt, assessment of quality

## Abstract

Background

Antigen rapid diagnostic testing (Ag-RDT) for severe acute respiratory syndrome coronavirus 2 (SARS-CoV-2) is an important testing technique used for the control and containment of COVID-19. This study aims to assess the quality of COVID-19 Ag-RDT in the testing sites in Ekiti State, Nigeria.

Methods

A validated World Health Organization (WHO) questionnaire was used to collect data from 138 testing sites located in 138 health facilities in Ekiti State. The assessment was based on the activities carried out in the last three months before the study.

Results

A total of 138 testing sites participated in the study out of which 121 (87.7%) were primary health facility testing sites. The mean number of samples tested in these sites was 26 samples, and 97 (70.3%) testing sites were tested below this. The average quality performance of the secondary/tertiary health facility testing sites (64.46 ± 10.47) was significantly higher than that of the primary health facility testing sites (53.13 ± 13.54) (p = 0.002). Additionally, the average quality performance of testing sites that tested 26 samples or more (61.61 ± 9.84) was significantly higher than that of the testing sites that tested below this mean cut-off (51.53 ± 13.97) (p < 0.001).

Conclusion

The majority of the testing sites that tested below the mean 26 samples, secondary/tertiary health facility testing sites, and sites that tested above the mean cut-off had higher average quality performance scores. Therefore, encouraging clinicians to refer for more testing of suspected cases and supportive supervision of COVID-19 Ag-RDT, especially in primary health facilities, is recommended.

## Introduction

Coronavirus disease 2019 (COVID-19) is a new disease that was detected when the Chinese authorities and the World Health Organization (WHO) became aware of a cluster of pneumonia cases with unknown etiology in Wuhan in December 2019 [1-3]. The disease was declared to be caused by severe acute respiratory syndrome coronavirus 2 (SARS-CoV-2) in January 2020 and was declared a disease of public health emergency of international concern (PHEIC) [1,2,4]. The virus belongs to the beta-corona virus genera and preferentially infects cells of the respiratory tract, but its organotropism to areas such as the brain, conjunctiva, pharynx, lungs, heart, liver, and kidney is not well understood [5].

COVID-19 has continued to be a worldwide challenge. Contact reduction and tracing, quarantine, vaccination, early detection, and treatment are key strategies to reduce the spread of the disease [6]. Testing remains a clinical tool, and it is a part of the first-line defense against COVID-19 as it enables early identification and isolation of cases, which helps slow down the disease transmission and helps target clinical care to those infected with overall protection of the health system [7,8].

At the inception of COVID-19 disease, the WHO recommends nucleic acid amplification test (NAAT) for identification of COVID-19 infection, but the test requires sophisticated laboratory infrastructures and skilled personnel [1,3]. Taking a nasopharyngeal swab, followed by a transcription-polymerase chain reaction of extracted ribonucleic acid is the recommended gold standard for the detection of SARS-CoV-2 [5]. Though numerous COVID-19 testing using the above technique have been performed globally, the demand for testing a large number of patients, more timely testing, and more accurate testing continues to exceed the testing capacity [3,5]. Also, technicality and the financial burden remain a challenge for this testing technique [5]. Furthermore, with the situation in the sub-Saharan African and low-income countries where molecular testing is mainly available in central reference laboratories with limited testing capacity, there emerged the need for a test technique to deal with the constraints of testing capacity, reduce backlogs, and ensure that patients with urgent clinical needs are prioritized [1,9].

Antigen rapid diagnostic testing (Ag-RDT) for SARS-CoV-2 is seen as an important testing technique to fight the spread of COVID-19 infection [3]. The Ag-RDT works by detecting antigens, and its accuracy depends on factors such as viral load in the specimen, quality of the sample, and the time from the onset of the infection [8]. Though the sensitivity of Ag-RDT is lower than NAAT, it has the advantages of lower cost and rapid result output [1,8]. The Ag-RDT is more simple, user-friendly, equipment-free, and can be operated safely at the point of care; the result is usually straightforward and quick [1,5,8].

Rapid diagnostic test supports the control of infection in both hospital and community settings [5]. It will allow expansion of testing access, enable accurate estimation of disease burdens, and help in achieving the targets of control measures and treatments [2,7]. The Ag-RDT for COVD-19 has been in operation in the Ekiti State, and this study aims to assess the quality of testing in the testing sites of Ekiti State, Nigeria.

## Materials and methods

This survey was a cross-sectional study carried out in Ekiti State. This state is one of the 36 states in Nigeria and is located in the southwestern part of the country. Ekiti State was carved out of the old Ondo State in October 1996 with the headquarters located in Ado-Ekiti. It has three senatorial districts (Ekiti Central, Ekiti South, and Ekiti North senatorial districts) divided into 16 local government areas (LGAs). The state has an estimated population of 2,384,212 (National Population Commission figures of 2006), with a 2021 projection of 3,816,784 based on an annual growth rate of 3.2% [10].

The testing sites are located in health facilities in all the senatorial districts and LGAs of the state. There were 141 testing sites located in 141 health facilities across the state at the time of this study, but only 138 of these testing sites/health facilities could be visited and assessed. The other three sites were not assessed due to security challenges in areas where they were located at the time when the survey was ongoing. The laboratory technicians at the testing sites were interviewed using a validated, interviewer-administered structured questionnaire between the 26th and 30th of August, 2021, and the assessment was based on the activities carried out in the last three months before the study. The content of the questionnaire was adopted from the Stepwise Process for Improving the Quality SARS-CoV-2 Antigen Rapid Diagnostic Testing (SPI-RT) checklist, which is based on the WHO assessment tool for laboratories implementing COVID-19 virus testing (Interim guidance: October 2020), and its format and layout were adapted from the SPI-RT checklist (stepwise) [11]. The questionnaire has sections on characteristics of the testing site, documents and records, personnel training and certification, safety, physical infrastructure, pre-testing phase, testing phase, post-testing phase, and external quality assessment (EQA).

Medical doctors and laboratory scientists were trained by the researchers for four hours a day on the data collection tools, techniques, and procedures as research assistants. The questionnaire was uploaded on open data kit (ODK) software and was used in the collection of data. The ODK had a global positioning system (GPS) tracker on it to ascertain that the research assistants visited the testing sites.

Data collected was downloaded from the ODK server created for the survey and later analyzed using the Statistical Package for the Social Science (SPSS) for Windows version 23.0 (IBM Corp., Armonk. NY). The questionnaire contains questions under each section with options of “yes,” “partial,” or “no.” Questions marked “yes” receive one point each; those marked “partial” receive 0.5 points each, and those marked “no” receive zero points each. However, some questions under the section of EQA were marked with “not applicable” (“NA”) in addition to “yes,” “partial,” and “no.” For every item marked “NA” in this section, the equivalent points are deducted from the expected total possible points. Total points scored for each section were tallied and recorded at the end of the section and expressed as a percentage of the total possible score of that section. The overall average percentage score from all eight sections was obtained as the average quality performance score for the testing sites.

Testing sites were grouped based on testing capacity and also based on the type of healthcare facility where they are located (primary, secondary, and tertiary). The tertiary healthcare facility was later grouped with secondary health facilities during the analysis because there was only one tertiary health facility with a testing site. Frequency, percentages, mean, and standard deviation were presented in tables at the univariate level of analysis. An independent student t-test was used to compare the average quality score between the two groups. p < 0.05 was taken as the significant value.

Ethical approval was sought and obtained from the Research and Ethics Review Committee of Federal Teaching Hospital, Ido-Ekiti, Ekiti State, Nigeria (Protocol number: ERC/2021/08/04/694B). Permission and consent for the study were taken from the heads of all the health facilities involved in the study.

## Results

Types of testing sites and average numbers of samples collected

A total of 138 testing sites participated in the study. Table 1 shows that out of the 138 testing sites, 121 (87.7%) were located in primary health facilities, 16 (11.6%) were located in secondary health facilities, and one (0.7%) was located in a tertiary health facility. The mean number of samples tested by the sites was 26 samples. More than two-thirds (70.3%) of the testing sites were tested below the mean sample cut-off, while 41 (29.7%) testing sites tested 26 samples and above.

**Table 1:** Type of testing sites and average number of samples tested

Variables	Frequency, N = 138	Percent (100%)
Types of testing sites		
Primary health facilities	121	87.7
Secondary health facilities	16	11.6
Tertiary health facilities	1	0.7
Mean number of samples tested (26 samples)		
Tested below 26 samples	97	70.3
Tested 26 samples and above	41	29.7

Quality assessment of testing sites

Table 2 shows the quality assessment scores of the testing sites. The average quality performance ± standard deviation of all the testing sites was 54.52 ± 13.65. The section with the highest quality score was the post-testing phase (74.15 ± 14.19) followed by the testing phase (67.03 ± 21.01). The lowest score was in the section on documents and records (32.95 ± 18.11).

**Table 2:** Quality assessment of testing sites

Sections	Average score ± standard deviation, N = 138
Documents and records	32.95 ± 18.11
Personnel training and certification	66.81 ± 32.71
Physical infrastructure	51.10 ± 23.56
Safety	48.08 ± 17.73
Pre-testing phase	66.05 ± 25.09
Testing phase	67.03 ± 21.01
Post-testing phase	74.15 ± 14.19
External quality assessment	30.02 ± 18.93
Average quality performance	54.52 ± 13.65

Relationship between the testing sites' characteristics and quality assessment

Table 3 shows that the average quality performance of the secondary/tertiary health facility testing sites (64.46 ± 10.47) was higher than that of the primary health facility testing sites (53.13 ± 13.54) (p = 0.002). The secondary/tertiary health facility testing sites had a significantly higher average score than the primary health facility testing sites in sections of documentation and records (43.95 ± 24.70 versus 31.71 ± 16.57, p = 0.010), physical infrastructure (74.35 ± 20.67 versus 47.78 ± 22.13, p < 0.001), safety (61.56 ± 16.20 versus 46.07 ± 17.08, p = 0.001), pre-testing phase (83.04 ± 15.18 versus 63.52 ± 25.20, p = 0.003), post-testing phase (81.25 ± 11.18 versus 73.05 ± 14.25, p = 0.029), and EQA (40.62 ± 22.64 versus 28.36 ± 17.86, p = 0.014). However, the secondary/tertiary health facility testing sites had lower average scores than primary health facility testing sites in sections of personnel training and certification (47.78 ± 29.88 versus 67.02 ± 33.31, p = 0.873) as well as the testing phase (65.28 ± 23.08 versus 67.54 ± 20.66, p = 0.686), but these differences were not statistically significant.

**Table 3:** Relationship between the testing sites’ characteristics and quality assessment SD: Standard deviation

	Characteristics of testing sites					
Sections	Types of testing sites			Mean number of samples tested		
	Primary health facilities (n = 121)	Secondary/tertiary health facilities (n = 17)		Tested below 26 samples (n = 97)	Tested 26 samples and above (n = 41)	
	Average score ± SD	Average score ± SD	T-test (p-value)	Average score ± SD	Average score ± SD	T-test (p-value)
Documents and records	31.71 ± 16.57	43.95 ± 24.70	-2.603 (0.010)	31.41 ± 16.38	36.59 ± 21.46	-1.541 (0.126)
Personnel training and certification	67.02 ± 33.31	47.78 ± 29.88	0.160 (0.873)	66.70 ± 35.90	67.07 ± 23.90	-0.061 (0.952)
Physical infrastructure	47.78 ± 22.13	74.35 ± 20.67	-4.545 (<0.001)	43.94 ± 22.23	68.04 ± 17.22	-6.195 (<0.001)
Safety	46.07 ± 17.08	61.56 ± 16.20	-3.428 (0.001)	45.46 ± 18.06	54.27 ± 15.43	-2.728 (0.007)
Pre-testing phase	63.52 ± 25.20	83.04 ± 15.18	-3.020 (0.003)	61.86 ± 25.98	75.96 ± 19.81	-3.112 (0.002)
Testing phase	67.54 ± 20.66	65.28 ± 23.08	0.406 (0.686)	65.92 ± 21.65	69.65 ± 21.65	-0.952 (0.343)
Post-testing phase	73.05 ± 14.25	81.25 ± 11.18	-2.212 (0.029)	71.25 ± 14.59	81.03 ± 10.50	-3.885 (<0.001)
External quality assessment	28.36 ± 17.86	40.62 ± 22.64	-2.500 (0.014)	25.70 ± 15.03	40.24 ± 23.08	-4.393 (<0.001)
Average quality performance	53.13 ± 13.54	64.46 ± 10.47	-3.218 (0.002)	51.53 ± 13.97	61.61 ± 9.84	-4.195 (<0.001)

Furthermore, the average quality performance of the testing sites that tested the mean 26 samples and above (61.61 ± 9.84) was higher than the testing sites that tested below the mean sample (51.53 ± 13.97) (p < 0.001). The testing sites that tested the mean 26 samples and above had a significantly higher average score than those that tested below the mean sample in sections of physical infrastructure (68.04 ± 17.22 versus 43.94 ± 22.23, p < 0.001), safety (54.27 ± 15.43 versus 45.46 ± 18.06, p = 0.007), pre-testing phase (75.96 ± 19.81 versus 61.86 ± 25.98, p = 0.002), post-testing phase (81.03 ± 10.50 versus 71.25 ± 14.59, p < 0.001), and EQA (40.24 ± 23.08 versus 25.70 ± 15.03, p < 0.001). However, there was no significant difference in the section on documentation and records, personnel training and certification, and testing phase between the testing sites that tested above the mean sample and below the mean sample.

## Discussion

COVD-19 Ag-RDT testing mainly occurred in primary healthcare facilities in comparison to the secondary and tertiary testing sites in the state. This may be because Ekiti State and Nigeria at large operate a ward health system where each ward has at least a primary health facility [12]. In addition, the requirement for the establishment of a primary health facility is much lesser than the secondary and tertiary health facilities. Primary healthcare facilities are also the first points of access to health care for the majority of inhabitants of communities.

More than three-quarters of the testing sites tested below the mean samples. This lower testing rate by the testing sites may be due to low patient patronage of the primary healthcare facilities as well as the community apathy toward COVID-19 testing as the majority of Ekiti inhabitants believed they were unlikely to get infected with COVID-19 [13].

The secondary/tertiary health facility testing sites did better than the primary health facility testing site in terms of the overall quality performance. This may be due to the availability of better infrastructures in terms of adequate space for testing, ventilation, and good lighting as well as better-skilled manpower in the secondary and tertiary health facilities than those in the primary health centers [14]. Also, the secondary/tertiary health facility testing sites were significantly better in sections of documentation and records, physical infrastructure, safety, pre-testing phase, post-testing phase as well as EQA. However, the primary health facility testing sites did better in the areas of the testing phase and personnel training and certification. Although this was not statistically significant, it may be because Ag-RDT testing is user-friendly, equipment-free, and easily operated at the point of care [1,5,8].

Furthermore, testing sites that tested above the testing mean cut-off did better in the quality assessment than those sites testing below the mean cut-off. The reason for this finding may be because testing sites that test above the mean cut-off have a better testing capacity and also get better with more testing. It was difficult to compare results as the literature search revealed no previous study that assesses the quality of Ag-RDT testing of COVID-19. COVID-19 is a new disease with so many uncertainties; however, several scientific research is still ongoing to unravel these. The limitation of this study was that all testing sites could not be assessed due to security compromises during the period of data collection.

## Conclusions

The majority of the testing sites tested below the mean 26 samples; secondary/tertiary health facility testing sites had better average testing performance than the primary health facility testing sites. Also, sites that tested more had higher average quality performance scores than those that tested less. Therefore, encouraging clinicians to refer for more testing and supportive supervision of COVID-19 Ag-RDT is recommended especially in primary health facility testing sites.

## Appendices

Stepwise process for improving the quality of SARS-CoV-2 antigen rapid diagnostic testing (SPI-RT) checklist (March 2021)

Introduction

The stepwise process for improving the quality of the SARS-CoV-2 antigen rapid diagnostic testing (SPI-RT) checklist is based on the WHO assessment tool for laboratories implementing COVID-19 virus testing (Interim Guidance: October 2020), and its format and layout were adapted from the SPI-RT checklist. It is intended to be used by supervisors, mentors, internal or external auditors undertaking testing site assessments or supervision visits in support of the implementation of quality assurance aspects of the SARS-CoV-2 antigen rapid testing. Site assessments or supervisions should be undertaken by trained persons that are responsible for supporting SARS-CoV-2 Ag-RDT rollout and implementation. The assessment findings should provide opportunities for improvement to SARS-COV-2 testing sites with the aim of ensuring the provision of quality COVID-19 Ag-RDT results. At a minimum, site assessments and/or supervision visits should be performed periodically, at least quarterly. However, more frequent visits should be arranged, particularly when a new test kit is introduced, a site is new, when there are new guidelines, or when many non-conformances are identified at the testing site.

In cases where the assessment report from the most recent visit is available, it should be reviewed prior to the supervision visit. During the visit, actions that were identified to address the areas of weakness during the previous visit must be reviewed and checked for completeness. After the support supervision, an on-site debriefing meeting should be conducted immediately before leaving the facility using the draft supervision report at hand. A final report, including supervision/assessment findings and the feedback from the debrief meeting and all relevant documents should be shared with the facility, Ministry of Health focal point, supporting implementing partner within one week. The supervised/assessed testing facility is required to develop and implement a corrective and preventive action plan to address all the non-conforming areas. In cases where the testing facility meets the required quality assurance requirements, effort should be made to maintain and sustain the quality management system.

Part A: Testing Facility Profile

Before completing the checklist, it is important to characterize the testing point to be audited. Please provide relevant information in the summary table below.

a) Date of Supervision (dd/mm/yyyy): ………/…………/…...…….

b) Supervision No. (where applicable): ……………………………..

c) Names & Affiliations of Officers conducting the site assessment/supervision/audit:

Name(s):                                                                  Title:                                                 Contacts (Affiliation, Tel, & Email)

…………………….………………………                       …………..…………….…………………                        …………………..………………….

…………………….………………………                       …………..…………….…………………                        …………………..………………….

d) Supervision Start Time:

e) Supervision End Time:

f) Testing Facility Name:

g) Testing Facility ID (if applicable):

h) Location (Physical Address of the Testing Facility):

Country: …………………….………………………                  District/Province: …………………….………………………      Street: …………………….………………………      

Postal Address: …………………….………………………      Email Address: …………………….………………………      

Telephone Contacts: …………………….………………………      

(Tick ü the correct option applicable to the Testing Facility being assessed/supervised)

i) Type of Testing Facility/Point

▢ Laboratory

▢ Care & Treatment Center

▢ Mobile Laboratory

▢ Point of Entry (PoC)

▢ Public Health Laboratories

▢ Prison

▢ School/Student Health Service

▢ Other (Specify): ………………….

j) Level of Testing Facility

▢ National Referral

▢ Regional Referral

▢ District Referral

▢ Health Center

▢ Dispensary

▢ Health Post

▢ Other (Specify): ………………….

k) Affiliation of Testing Facility

▢ Government

▢ Private for Profit

▢ Private Not for Profit

▢ Faith-Based Organization

▢ Non-governmental organization

▢ Other (Specify): ………………….

l) Name(s) of Testing Facility Staff Interviewed/Supervised:

           Name:                                                                Title:                                             Contacts (Tel & Email):

…………………….………………………                       …………..…………….…………………                        …………………..………………….

…………………….………………………                       …………..…………….…………………                        …………………..………………….

…………………….………………………                       …………..…………….…………………                        …………………..………………….

m) Number of testers at the testing facility (if applicable):

n) Number of COVID-19 Ag-RDT performed in the last consecutive months:

Tests performed (total test):

Number positive:

Number Invalid:

Month 1:

Month 2:

Month 3:

Average Number of Tests Performed Per Month:

Part B. Testing Facility Components for Assessment

For each of the sections listed below, please indicate a response Yes, Partial, or No against each requirement/question. Indicate “Yes” only when all elements are satisfactorily present. Provide comments for each “Partial” or “No” response. For each “Yes” response, award a score of one point in the adjacent cell; for Partial, award 0.5 points, and for No, a zero-point score is awarded. State N/A in the comments section if “not applicable” where appropriate (*).

**Table 4:** Documentation and records

4.0	Documentation and Records	Y	P	N	Comments
4.1	Are the following guidelines specific for SARS-CoV-2 antigen rapid diagnostic testing available at the testing facility? Instruction: The Evaluator/Auditor/Mentor/Supervisor must establish and determine the relevant latest International & National Guidelines before assessing the testing facility. For each guideline(s) listed below, award 1 point if the current version of the guideline is available, award 0 points if unavailable (and 0.5 points if available but not the current version or otherwise)				
a.	Africa CDC Interim Guidance on the Use of Rapid Antigen Tests for COVID-19 Response				
b.	Quality Assurance Framework for SARS-COV-2 antigen rapid testing for the diagnosis of COVID-19 (by the Africa CDC/ASLM)				
c.	WHO “Interim” Guidance antigen detection in the diagnosis of SARS-COV-2 infection using immunoassays (latest guidance)				
d.	Are there SOPs and/or job aides in place to implement safety practices?				
e.	Are there SOPs and/or job aides in place on how to dispose of infectious and non-infectious waste?				
f.	Are there SOPs and/or job aides in place to manage spills of COVID-19 samples, blood, and other body fluids?				
4.2	Is the national SARS-CoV-2 antigen rapid diagnostic testing algorithm available at the testing facility/site?				
4.3	Are there national guidelines describing how client identification should be recorded in the SARS-CoV-2 antigen rapid diagnostic testing register?				
4.4	For each of the SARS-CoV-2 Ag-RDT kits in use at the testing facility, are the manufacturer instructions/manuals/inserts available and accessible to testers?				
4.5	Are SOPs and/or job aides in place for each SARS-CoV-2 antigen rapid diagnostic test used in the testing algorithm available and posted at the testing point?				
4.6	Are national biosafety guidelines for COVID-19 for infection prevention and control available and accessible to the testers?				
4.7	Does the testing facility have national/regional guidelines for monitoring COVID-19 Ag-RDT key performance and quality indicators?				
4.8	Have the site performed and documented risk assessment according to the National Guidelines or WHO Interim Guidelines?				
4.9	Are there a national standardized SARS-CoV-2 Ag-RDT tests register/logbook available for recording test results?				
4.10	Are there records indicating all testers have demonstrated competency in SARS-CoV-2 antigen rapid diagnostic testing prior to client testing?				
4.11*	Are there records in the testing register/logbook for swapped results received from the referral lab?				N/A
	Documents & records scores attained				
	Documents & records maximum possible score	16			
	Documents & records % score attained instruction: % score computation = scores attained divided by maximum possible score x 100%				

**Table 5:** Personnel training and certification

5.0 Personnel Training and Certification		Y	P	N	Comments
5.1	Have all testers received a comprehensive training on COVID-19 Ag rapid testing?				
5.2	Are the testers trained on the use of standardized SARS-CoV-2 antigen rapid diagnostic test registers/logbooks?				
5.3	Are the testers trained on quality control (QC) processes?				
5.4	Are the testers trained on safety, risk assessment, and waste management procedures and practices?				
5.5	Are only certified testers allowed to perform the SARS-CoV-2 antigen rapid diagnostic test?				
	Personnel training & certification scores attained				
	Personnel training & certification maximum possible score	5			
	Personnel training & certification % score attained instruction: % score computation = scores attained divided by maximum possible score x 100%				

**Table 6:** Physical facility

6.0	Physical Facility	Y	P	N	Comments
6.1	Is there a designated area for SARS-CoV-2 antigen rapid diagnostic testing?				
6.2	Is the testing area clean and organized for SARS-CoV-2 antigen rapid diagnostic testing?				
6.3	Is sufficient lighting available in the designated testing area?				
6.4	Are the SARS-CoV-2 Ag-RDT kits stored within the temperature range based on the manufacturers’ instructions?				
6.5	Is there sufficient and secure storage space for test kits and other consumables?				
6.6	Is the space allocated for SARS-CoV-2 antigen rapid diagnostic testing adequate to perform the work without compromising the quality and safety of patients and testing personnel/health workers?				
6.7	Is the COVID-19 sample collection area separated from the patient examination/testing areas/room(s)?				
	Physical facility scores attained				
	Physical facility maximum possible score	7			
	Physical facility % score attained instruction: % score computation = scores attained divided by maximum possible score x 100%				

**Table 7:** Safety

7.0 Safety		Y	P	N	Comments
7.1	Is there a biohazard sign on the doors of the rooms/or at the designated area where COVID-19 RDT testing is done?				
7.2	Are the following personal protective equipment available in sufficient quantity at the testing facility? Instruction: For each of the listed PPE below, award 1 point if available in sufficient quantities, 0 points if unavailable and 0.5 points if available but in insufficient quantities				
a.	Gloves				
b.	Gowns/Laboratory coats				
c.	Eye protection or face shields				
d.	Respirator (FFP2 or N95)				
e.	Face masks				
7.3	Is PPE consistently and properly used by all testers through the testing process? Instructions: The evaluator/assessor/supervisor observes for consistent use of the PPE throughout the processes before scoring this requirement.				
7.4	Is there an installation/sink/facility dedicated to handwashing?				
7.5	Is there clean water and soap available for handwashing?				
7.6	Is an appropriate disinfectant to clean the work area available?				
7.7	Are leak-proof biohazard bags and appropriate waste bins available and properly used on the testing site?				
7.8	Are sharps, infectious, and non-infectious waste handled properly?				
7.9	Are infectious and non-infectious waste containers emptied regularly per the SOP and/or job aides?				
	Safety scores attained				
	Safety maximum possible score	13			
	Safety % score attained instruction: % score computation = scores attained divided by maximum possible score x 100%				

**Table 8:** Pre-testing phase

8.0 Pre-testing Phase		Y	P	N	Comments
8.1	Is the national SARS-CoV-2 antigen rapid diagnostic testing algorithm being used at the testing facility/point and adhered to?				
8.2	Is there a process in place for an alternative SARS-CoV-2 antigen rapid diagnostic testing algorithm in case of an expired or shortage of test kit(s)?				
8.3	Are only national or WHO Emergency Use Listing Procedure (EUL) approval SARS-CoV-2 Ag-RDT approved test kits available for use currently?				
8.4	Are all the test kits currently in use within the expiration date?				
8.5	Are the test kits labeled with the date received and initials?				
8.6	Are there sufficient supplies available for client sample collection?				
8.7	Are client identifiers recorded in the SARS-CoV-2 antigen rapid diagnostic testing register per national guidelines and on test devices?				
*8.8	If samples are shipped to a reference lab, does the laboratory have appropriate packaging for referring specimens (triple package if air transport, or any package in conformity with local regulations or recommendations)?				N/A
8.9	Is a transportation system for sample referral (bus, ambulance, national postal service, etc.) already set up?				
8.10	Is/are the person(s) in charge of shipments trained for the transport of infectious substances?				
	Pre-testing phase scores attained				
	Total of “Not Applicable”				
	Pre-testing phase maximum possible score	10			Guidance: In case, checklist item 5.8 is not applicable, then subtract 1 point from the total 10 points.
	Pre-testing phase % score attained instruction: % score computation = scores attained divided by maximum possible score x 100%				

**Table 9:** Testing phase

9.0 Testing Phase		Y	P	N	Comment
9.1	Are timers available and used routinely for SARS-CoV-2 antigen rapid diagnostic testing?				
9.2	Did the tester put on the appropriate PPE for testing?				
9.3	Are sample collection devices used correctly?				
9.4	Are testing procedures adequately followed?				
9.5	Is IQC performed and documented according to National/International Guidelines or kit manufacturer instructions?				
9.6	Did the tester put on the appropriate PPE for testing?				
9.7	Did the tester adhere to the manufacturer's instructions for using the SARS-CoV-2 antigen RDT?				
9.8	Did the tester have all the necessary supplies to perform the SARS-CoV-2 antigen RDT procedure before starting the sample testing process?				
9.9	Did the tester set up the workstation correctly?				
9.10	Did the tester check the expiry date of the SARS-CoV-2 antigen RDT?				
9.11	Did the tester check that the test device and the desiccant pack in the foil pouch were not damaged or invalid?				
9.12	Did the tester insert the swab into an extraction buﬀer tube and, while squeezing the buﬀer tube, stir the swab?				
9.13	Did the tester remove the swab while squeezing the sides of the tube to extract the liquid from the swab?				
9.14	Did the tester press the nozzle cap tightly onto the tube?				
9.15	Did the tester apply the required number of drops of the extracted specimen to the specimen well of the test device?				
9.16	Are incorrect/invalid QC results properly recorded?				
9.17	Are appropriate steps taken and documented when QC results are incorrect and/or invalid?				
9.18	Are QC records reviewed by the person in charge routinely?				
	Testing phase scores attained				
	Testing phase maximum possible score	18			
	Testing phase % score attained instruction: % score computation = scores attained divided by maximum possible score x 100%				

**Table 10:** Post-testing

10.0 Post-testing Phase		Y	P	N	Comments
10.1	Is a national standardized register/logbook being used correctly and consistently to record SARS-CoV-2 antigen RDT test results?				
10.2	Does the SARS-CoV-2 antigen RDT tests testing register/logbook include all of the key quality elements?				
10.3	Are all the elements in the register/logbook recorded/captured correctly? (e.g., client demographics, kit names, lot numbers, expiration dates, tester name, individual and final SARS-CoV-2 antigen RDT test results, etc.)?				
10.4	Is there documented evidence that clinicians are immediately notified of SARS-CoV-2 test positive results to inform timely patient isolation and management/treatment?				
10.5	Are invalid test results recorded in the register/logbook as well?				
10.6	Are invalid tests repeated and results properly recorded in the register/logbook?				
10.7	Are all client documents and records securely kept throughout all phases of the testing process?				
10.8	Are all registers/logbooks and other documents kept in a secure location when not in use?				
10.9	Are registers/logbooks properly labeled and archived when full?				
	Testing phase scores attained				
	Testing phase maximum possible score	9			
	Testing phase % score attained instruction: % score computation = scores attained divided by maximum possible score x 100%				

**Table 11:** External quality audit

11.0 External Quality Audit (PT, Supervision, and Retesting)		Y	P	N	Comments	
11.1	Is the testing facility enrolled in an EQA/PT program?					
11.2	Do all testers at the testing facility test the EQA/PT samples?					
11.3	Does the person in charge at the testing facility review the EQA/PT results before submission to NRL or the designee?					
11.4	Is an EQA/PT report received from National Reference Laboratory and reviewed by testers and/or the person in charge at the testing facility?					
11.5	Does the testing facility conduct sample retesting using other laboratories as an alternative to SARS-CoV-2 Ag-RDT EQA?					
11.6	Does the testing facility implement corrective action in case of unsatisfactory results?					
11.7	Does the testing facility receive periodic supervisory visits?					
11.8	Is feedback provided during the supervisory visit and documented?					
11.9	If testers need to be retrained, are they being retrained during the supervisory visit?					
					N/A	Guidance: In case checklist items 8.10-8.13, whichever is not applicable, subtract 1 point from the total 13 points for each item.
11.10*	Does the site collect swap samples for retesting according to country guidelines (e.g., collection of every 20^th^ client sample)?					
11.11*	Are the swap samples collected for retesting properly documented?					
11.12*	Are swap samples stored properly?					
11.13*	Are the identifier’s samples sent for retesting properly recorded?					
	External quality audit scores attained					
	External quality audit maximum possible score	13				
	External quality audit % score attained instruction: % score computation = scores attained divided by maximum possible score x 100%					
